# Efficacy and safety of indocyanine green fluorescence imaging in colorectal cancer: a systematic review and meta-analysis of randomized controlled trials

**DOI:** 10.1007/s00384-025-04941-7

**Published:** 2025-09-10

**Authors:** Abdullah Afridi, Ayesha Zulfiqar, Fatima Sajjad, Iqra Shahid, Hira Habib, Yasir Saleem, Zain Afridi, Asad Iqbal, Fazia Khattak, Farwa Nisa, Hanifullah Khan, Zaryab Bacha, Muhammad Abdullah Ali, Muhammad Hamza Khan, Rizwan Afridi, Kamil Ahmad Kamil

**Affiliations:** 1 Khyber Medical College, Peshawar, Pakistan; 2 Dow Medical College, Karachi, Pakistan; 3 King Edward Medical University, Lahore, Pakistan; 4 Allama Iqbal Medical College, Lahore, Pakistan; 5 Bacha Khan Medical College, Mardan, Pakistan; 6 Department of Medicine, Fatima Jinnah Medical University, Lahore, Pakistan; 7 Department of Surgery, Khyber Teaching Hospital, Peshawar, Pakistan; 8 Khyber Teaching Hospital, Peshawar, Pakistan; 9 Internal Medicine Department, Mirwais Regional Hospital, Kandahar, Afghanistan

**Keywords:** Colorectal cancer, Randomized controlled trials, Meta-analysis, Indocyanine green fluorescence imaging

## Abstract

**Background:**

The primary treatment for colorectal cancer, which is very prevalent, is surgery. Anastomotic leaking poses a significant risk following surgery. Intestinal perfusion can be objectively and instantly assessed with indocyanine green fluorescence imaging, which may lower leakage rates and enhance surgical results.

**Methods:**

PubMed, Embase, and Web of Science databases were systematically searched using relevant keywords from inception until 5th of March 2025. Eight studies were included after final screening. Outcomes were reported as overall anastomotic leakage, wound infection, paralytic ileus, mechanical ileus, and post-operative hospital stay. Interstudy heterogeneity was assessed using *I*^2^ and *X*^2^ statistics (*I*^2^ > 50% = significant heterogeneity). Statistical calculations were performed using Review Manager 5.4.1 (The Cochrane Collaboration, Copenhagen, Denmark), with a *p*-value of < 0.05 indicating statistical significance.

**Results:**

This meta-analysis includes 4047 patients from eight investigations (2026 indocyanine green (ICG) group, 2021 non-ICG group). Overall anastomotic leak risk was considerably decreased with ICG use (risk ratio (RR) = 0.66; 95% CI: 0.54–0.81; *p* < 0.0001) and showed no heterogeneity (*I*^2^ = 0%). There was no heterogeneity (*I*^2^ = 0%) in the Grade A leakage occurrence, which was considerably lower in the ICG group (RR = 0.34; 95% CI: 0.16–0.72; *p* = 0.005). With little heterogeneity (*I*^2^ = 8.6%), combined leakage grades also supported ICG use (RR = 0.54; 95% CI: 0.35–0.84; *p* = 0.006). ICG was associated with a substantial decrease in Clavien–Dindo Grade I complications (RR = 0.67; 95% CI: 0.49–0.92; *p* = 0.01) without heterogeneity (*I*^2^ = 0%). Initial postoperative hospital stays, mechanical ileus, paralytic ileus, and abdominal bleeding did not differ significantly. Although there was no heterogeneity (*I*^2^ = 0%), sensitivity analysis showed that the ICG group had a substantially longer postoperative stay (MD = 0.27; 95% CI 0.05–0.49; *p* = 0.02) and significantly fewer wound infections (RR = 0.17; 95% CI 0.04–0.76; *p* = 0.02). With noteworthy heterogeneity (*I*^2^ = 70%), the ICG group’s operating time was significantly longer (MD = 8.26 min; 95% CI 0.52–16.00; *p* = 0.04).

**Conclusion:**

Although indocyanine green fluorescence imaging may marginally lengthen the recovery period and duration of operation, it dramatically lowers anastomotic leakage and wound infections following colorectal surgery, enhancing results.

**Supplementary Information:**

The online version contains supplementary material available at 10.1007/s00384-025-04941-7.

## Introduction

With approximately 2.2 million new cases and 1.1 million deaths in 2019, colorectal cancer is the second most frequent cancer worldwide [1]. Of these patients, the rectum is where about one-third of all colorectal malignancies are found [2]. The preferred course of treatment for colorectal cancer is surgery, and sufficient lymph node dissection is necessary for this procedure. Accurate colorectal cancer staging can be achieved by extracting at least *1*^2^ lymph nodes, according to the AJCC guidelines [3]. Patients (2.8 to 19%) experience anastomotic leakage, a serious side effect after colon-rectal surgery [4]. It not only has a major influence on a patient’s overall quality of life but also plays a crucial role in predicting their prognosis [5, 6]. Clinical evaluation of anastomotic leak risk is difficult, even for skilled surgeons [7]. Although the etiology of AL is complex, inadequate perfusion is thought to be a major contributing component to the pathophysiology [8, 9].

A variety of techniques have been put forth to evaluate adequate intestinal perfusion objectively in addition to the surgeon’s subjective assessment during the procedure [10]. With the use of laparoscopic instruments with specialized electronic filters, intraoperative fluorescent angiography (FA) employing indocyanine green (ICG) is a technique that enables one to evaluate blood flow through the intestinal composite sections and thereby avoid the occurrence of postoperative anastomotic leakage [11, 12]. An excellent method for determining tissue perfusion is to use indocyanine green (ICG), a water-soluble fluorescent dye that binds to plasma proteins. ICG absorbs and releases near-infrared light, which contemporary laparoscopic cameras and other specialized near-infrared fluorescence imaging equipment can see. Surgeons have historically evaluated the viability of anastomosis based on arterial bleeding from the transected end of the colon and bowel color. For over 10 years, ICG fluorescence imaging has been employed to improve perfusion assessment at intended anastomotic locations [13].

This systematic review and meta-analysis aims to pool evidence from RCTs to evaluate the efficacy and safety of ICG in improving intraoperative and postoperative outcomes. Despite encouraging data, there are currently no established procedures for using ICG in colorectal surgery, and more research is necessary to determine its precise effect on lowering anastomotic leakage.

## Methods

### Study design and protocol registration

This systematic review was in accordance with the guidelines set by the Cochrane Collaboration and the Preferred Reporting Items for Systematic Reviews and Meta-Analysis (PRISMA) framework [14]. It encompassed the study design, stepwise implementation, analysis, and presentation of findings. Additionally, the study protocol was registered in the International Prospective Register of Systematic Reviews (PROSPERO) under registration number** (**CRD420251041194).

### Search strategy and databases

A comprehensive electronic search was conducted across PubMed, Embase, and Web of Science, including all available records from their inception up till 5th of March 2025, with no language restrictions. The search was conducted using the following keywords: “colorectal cancer,” “colorectal anastomotic leakage,” and “indocyanine green fluorescence imaging”. To enhance the validity and generalizability of our review, a supplementary search was performed by thoroughly reviewing references of all incorporated studies, thereby uncovering additional articles that may have been excluded.

### Study selection and eligibility criteria

All studies identified through the online search were imported into the Rayyan software for screening, where duplicate entries were removed. The remaining articles underwent an initial review based on their titles and abstracts. Full-text articles were retrieved if either investigator deemed the abstracts relevant. Two reviewers (YS and AA) independently assessed each study’s eligibility according to the inclusion criteria, with any disagreements resolved through discussion with a third researcher (ZB). The studies included in this systematic review met the following criteria: (1) RCTs, (2) inclusion of patients having colorectal cancer, (3) interventions involving indocyanine green fluorescence imaging, (4) non-indocyanine green as control, and (5) reporting at least three relevant outcome. The exclusion criteria included (1) overlapping populations, identified by shared institutions and recruitment periods; (2) populations not within the scope of interest; (3) republished studies; (4) protocols without reported results; (5) observational studies reviews, abstracts, case reports, case series, background articles, expert opinions, or in vivo/in vitro studies; (6) duplicate data from the same clinical trial; and (7) lack of a comparator group.

### Data extraction and outcomes

Two authors (HH and AA) independently extracted data using a predefined Microsoft Excel spreadsheet. Any discrepancies during the extraction process were resolved by a third author (ZB). Data were collected from study text, tables, and figures, with raw values estimated from percentages when necessary. Study and patient characteristics included (country, age, BMI sample size, current smokers, tumor type, surgery, diabetes, chemotherapy, radiotherapy, ASA score, and AJCC score)*.* The primary outcomes of the study included overall anastomotic leak; anastomotic leakage occurrence Grade A, B, and C; and Clavien–Dindo scale, while the secondary outcomes included abdominal bleeding, complications, mechanical ileus, paralytic ileus, wound infection, operating time, and post-operative hospital stay. See Supplementary Table (Table S1) for detailed definition of outcomes.

### Quality assessment

The quality assessment of the included studies was conducted using appropriate tools based on their study design. For randomized controlled trials (RCTs), the Revised Cochrane Risk of Bias Tool for Randomized Trials (RoB 2) [15] was applied, evaluating bias across five key domains: the randomization process, deviations from intended interventions, missing outcome data, outcome measurement, and selection of reported results. Each study’s overall risk of bias was classified as low, some concerns, or high risk. This tool is the standard tool employed for assessing the risk of bias and quality of the included studies and is used by various meta-analyses [16]. Two reviewers (ZA and YS) independently evaluated the risk of bias, resolving any disagreements through discussion. If necessary, a third reviewer (AA) was consulted for consensus. This systematic assessment helped ensure the reliability and validity of the included studies.

### Certainty of evidence

The Grading of Recommendations, Assessment, Development, and Evaluation (GRADE) approach was used by two independent authors (AI and AA) with the GRADEpro Guideline Development Tool [17] to assess the certainty of evidence in this meta-analysis. The evidence was classified into four levels: high, moderate, low, or very low [18]. Any disagreements were resolved through discussion and consensus. Other meta analyses also report using the GRADE approach for assessing the quality of evidence [19].

### Statistical analysis

All statistical analyses were performed using RevMan (version 5.4; Copenhagen: The Nordic Cochrane Centre, The Cochrane Collaboration, 2014) [20]. The results were visualized in forest plots. Binary outcomes were analyzed using risk ratios (RRs), while continuous outcomes were assessed with mean differences (MDs), both presented in forest plots. Heterogeneity was evaluated using the Cochrane *Q* chi-square test and the *I*^2^ statistic, with *P*-values < 0.10 and *I*^2^ > 50% indicating significant heterogeneity [21]. To test the robustness of the pooled estimates, statistical analysis was performed, sequentially removing each study and reanalyzing the data to ensure that no single study disproportionately influenced the overall effect sizes.

## Results

### Searched results

**Fig. 1:**
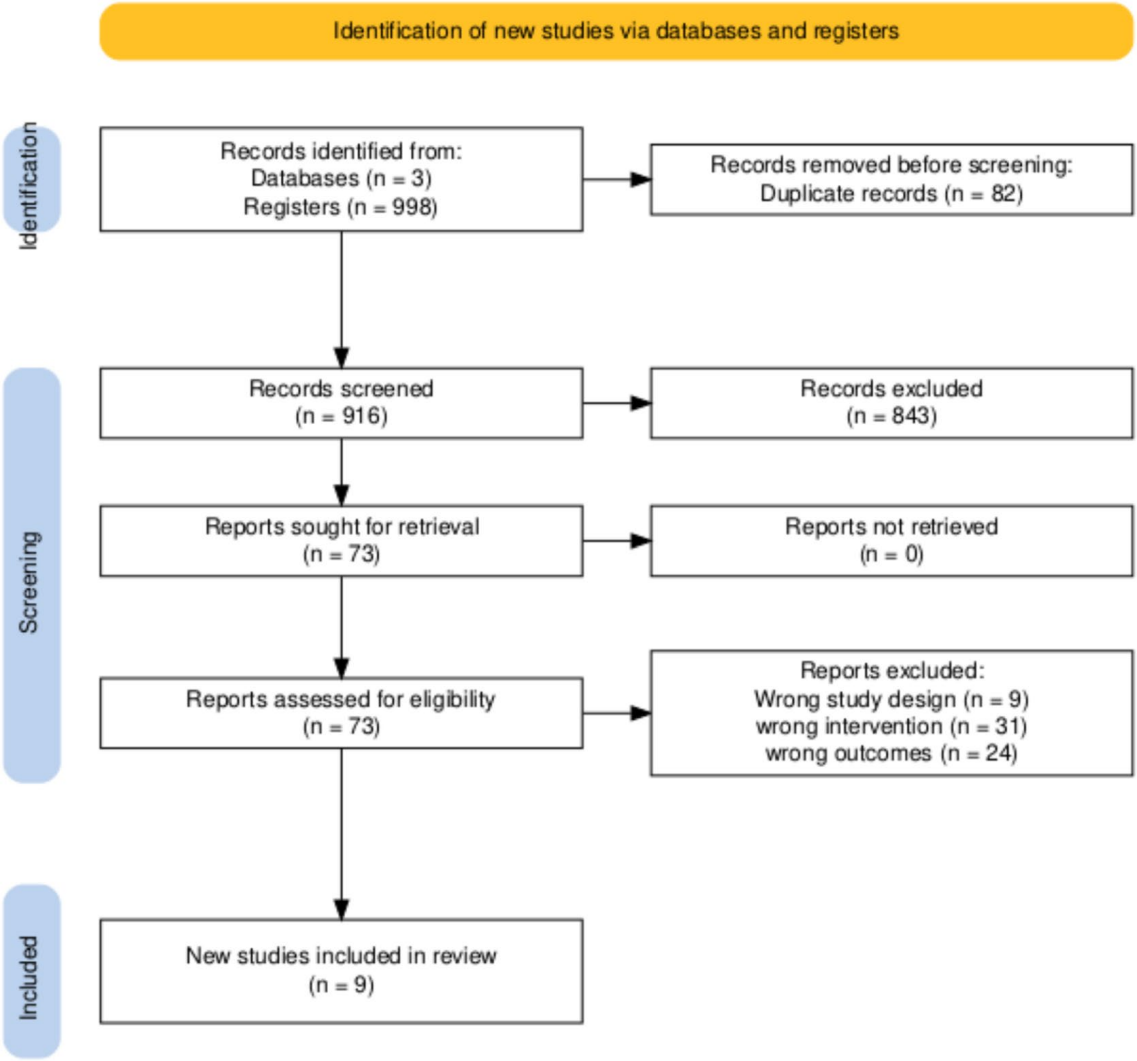
Prisma flowchart

A total of 998 records of the articles were retrieved from three databases; 723 from PubMed, 148 from Web of Science, and 127 from Embase using a search string including all the relevant MeSH terms. After removal of duplicates, 916 articles were remaining for title and abstract screening. Following the screening process, 843 articles were excluded and 73 were assessed for eligibility. Nine articles were included in our meta-analysis. Details of the screening process are given in Fig. 1**.**

### Study characteristics

Our meta-analysis included nine randomized controlled trials [13, 22–29] involving a total of 4054 patients, with 2028 in the ICG group and 2026 in the non-ICG group. The average age of participants was 63.9 years. The proportion of male participants was 109 in ICG group and 106 in the non-ICG group. The studies were conducted in Russia, Egypt, Poland, US, Italy, Netherland, China, and Japan. The mean BMI was 25.4 kg/m^2^ in the ICG group and 25.6 kg/m^2^ in the non-ICG group. The proportion of current smokers was 13% in both groups. The prevalence of diabetes was 12% in the ICG group and 13% in the non-ICG group. Benign tumors were observed in 19% of patients in both groups, while malignant tumors were present in 81% of cases in each group. Detailed baseline characteristics is mentioned in Tables 1 and 2**.**

**Table 1:** Study characteristics

Author, year	Recruitment period	Country	Type of study	Intervention	Control	ICG dose and route of administration	Height of anastomosis from anal verge	Sample size		Male, *N* (%)	
								ICG	Non-ICG	ICG	Non-ICG
Alekseev, 2020	Nov 2017 to Aug 2019	Russia	RCT	ICG	Non-ICG	0.2 mg/kg Intravenous	between 4 and 8 cm from anal verge	187	190	92 (49)	92 (48)
Eltaweel, 2024	Jan 2022 to Oct 2022	Egypt	RCT	ICG	Non-ICG	0.3 mg/kg Intravenous	2 to 15 cm from the anal verge	50	51	25 (50)	28 (54.9)
Gach, 2023	Dec 2020 to Aug 2021	Poland	RCT	ICG fluorescent angiography	Non-ICG	1 ampoule (50 mg) Intravenous	up to 12 cm from the anal verg**e**	41	35	26 (63.4)	21 (60)
Jafari, 2021	Mar 2015 to Feb 2017	US	RCT	ICG	Non-ICG	3.0 ± 1.0 mL of a 2.5-mg/mL Intravenous	≤ 10 cm from the anal verge	178	169	108 (61.2)	99 (58.6)
Nardi, 2019	Jan 2016 to Nov 2017	Italy	RCT	ICG	Non-ICG	0.3 mg/kg Intravenous	Anastomosis between 2 and 15 cm from the anal verge	118	122	60 (50.8)	66 (54)
Rinne, 2025	Sept 2018 to Dec 2023	US	RCT	ICG	Non-ICG	5 mg Intravenous	upper third of the rectum	536	541	297 (55.4)	284 (52.5)
Robin, 2024	Jul 2020 to Feb 2023	Netherlands	RCT	FGBA	CBA	5 mg Intravenous	NR	463	468	249 (54)	236 (50)
Wan, 2022	May 2021 to Apr 2022	China	RCT	ICG	Non-ICG	25 mg of ICG powder dissolved in 10 mL of sterile water through Endoscopic submucosal injection	NR	33	33	21 (63.6)	24 (72.7)
Watanabe, 2023	Dec 2018 to Feb 2021	Japan	RCT	ICG	Non-ICG	12.5 mg through intravenous	lower margin located < 12 cm from the anal verge	422	417	NR	NR

*ICG* indocyanine green, *FGBA* fluorescent guided bowel anastomosis, *CBA* conventional bowel anastomosis

**Table 2:** Patient characteristics

	Alekseev 2020		Eltaweel 2024		Gach 2023		Jafari 2021		Nardi 2019		Rinne 2025		Robin 2024		Wan 2022		Watanabe 2023	
	ICG	Non-ICG	ICG	Non-ICG	ICG	Non-ICG	ICG	Non-ICG	ICG	Non-ICG	ICG	Non-ICG	ICG	Non-ICG	ICG	Non-ICG	ICG	Non-ICG
Age (yrs) Mean (SD)	56 (48)	71 (16)	66.1 (12)	65.1 (11)	64.7 (10)	64.8 (10)	57.2 (11)	57.0 (11)	66.1	65.1	70 (11)	70 (11)	67 (11)	67 (12)	58 (30)	60 (27)	NR	NR
BMI (kg/m^2^) Mean (SD)	NR	NR	25 (7.2)	25 (8.7)	26 (4.7)	26 (4.2)	27 (5.6)	28 (5.9)	25.2	25.6	27 (5)	27 (5)	26 (4.2)	26 (4.1)	22 (10)	22 (8.6)	NR	NR
Current smokers *N* (%)	NR	NR	NR	NR	NR	NR	48 (27)	30 (18)	NR	NR	55 (10)	63 (11)	49 (11)	59 (13)	NR	NR	NR	NR
Tumor type *N* (%)																		
Benign		6 (3.2)	14 (28)	15 (29)	NR	NR	NR	NR	32 (27)	37 (30)	156 (29)	158 (29)	58 (13)	47 (10)	NR	NR	NR	NR
Malignant	183 (97)	184 (96)	36 (72)	36 (70)	NR	NR	NR	NR	86 (72)	85 (69)	380 (70)	383 (70)	405 (87)	421 (90)	NR	NR	NR	NR
Type of surgery *N* (%)	LC: 17 (9.1) AR: 59(32) LAR: 111 (59)	LC: 25 (13) AR: 60 (32) LAR: 105 (55)	LC: 26 (52) LAR: 24 (48)	LC: 29 (56) LAR: 22 (43)	NR	NR	NR	NR	LC: 62 (52) LAR: 56 (47)	LC: 69 (56) LAR: 53 (43)	NR	NR	IR: 11 (2) RH: 178 (38) TC: 4 (1) LH: 42 (9) SigC: 132 (29) LAR: 88 (19) SC: 6 (1) TTME: 2 (< 1)	IR: 15 (3) RH: 193 (41) TC: 3 (1) LH: 39 (8) SigC: 122 (26) LAR: 96 (21) SC: 0 TTME: 0	NR	NR	NR	NR
Diabetes *N* (%)	15 (8.0)	17 (8.9)	9 (18)	11 (21)	NR	NR	22 (12)	22.9 (13)	17 (14)	19 (16)	NR	NR	60 (13)	62 (13)	NR	NR	NR	NR
Chemotherapy *N* (%)	NR	NR	14 (28)	12 (23)	NR	NR	17 (9.6)	10 (6.4)	27 (22)	21 (17)	NR	NR	7 (2)	5 (1)	NR	NR	NR	NR
Radiotherapy *N* (%)	NR	NR	14 (28)	12 (23)	NR	NR	113 (63)	111 (65)	28 (23)	21 (17)	NR	NR	10 (2)	14 (3)	NR	NR	NR	NR
ASA score *N* (%)																		
1	18 (9.6)	22	4 (8)	3 (5.8)	1 (2.4)	0	NR	NR	10 (8.4)	7 (5.7)	26 (4.9)	33 (6.1)	NR	NR	1 (3)	1 (3)	NR	NR
2	145 (77)	143	35 (70)	38 (74)	23 (56)	16 (45)	NR	NR	82 (69)	92 (75)	249 (46)	224 (41)	NR	NR	29 (87)	30 (90)	NR	NR
3	24 (12)	25	11 (22)	10 (19)	17 (41)	18 (51)	NR	NR	26 (22)	23 (18)	237 (44)	255 (47)	NR	NR	3 (9.1)	2 (6.1)	NR	NR
UICC/AJCC stage *N* (%)																		
0	3 (1.6)	7 (3.8)	NR	NR	3 (7.3)	3 (8.6)	NR	NR	NR	NR	NR	NR	NR	NR	NR	NR	NR	NR
I	37 (20)	25 (13)	NR	NR	13 (31)	11 (31)	NR	NR	NR	NR	70 (19)	82 (22)	NR	NR	6 (18)	4 (12)	NR	NR
II	49 (26)	46 (25)	NR	NR	6 (14)	9 (25)	NR	NR	NR	NR	140 (39)	132 (36)	NR	NR	15 (45)	17 (51)	NR	NR

*ICG* indocyanine green, *ASA* American Society of Anesthesiologists, *AJCC* American Joint Committee on Cancer, *LC* left colectomy, *LAR* low anterior rectal resection, *AR* anterior resection, *IR* ileocaecal resection, *RH* right hemicolectomy, *TC* transversectomy OR transverse colectomy, *LH* left hemicolectomy, *SigC* sigmoidectomy, *LAR* low anterior resection, *SC* subtotal colectomy, *TTME* transanal total mesorectal excision, *AV* anal verge

### Risk of bias

Five of the included studies were assessed as having a low risk of bias, while four studies were rated as having some concerns. One study [25] exhibited some concerns in Domain 1 (bias arising from randomization process), three studies [25–27] showed some concerns in Domain 2 (bias due to deviation from intended interventions), and two studies [23, 25] were known to have some concerns in Domain 4 (bias in the measurement of outcome). A detailed assessment of the risk of bias is presented in Supplementary Figure S1.

### Certainty of evidence

The GRADE approach, using the GRADEpro Guideline Development Tool, was employed to assess the certainty of evidence. A detailed assessment is shown in Table S2.

### Outcomes

#### Primary outcomes

##### Overall anastomotic leak

**Fig. 2:**
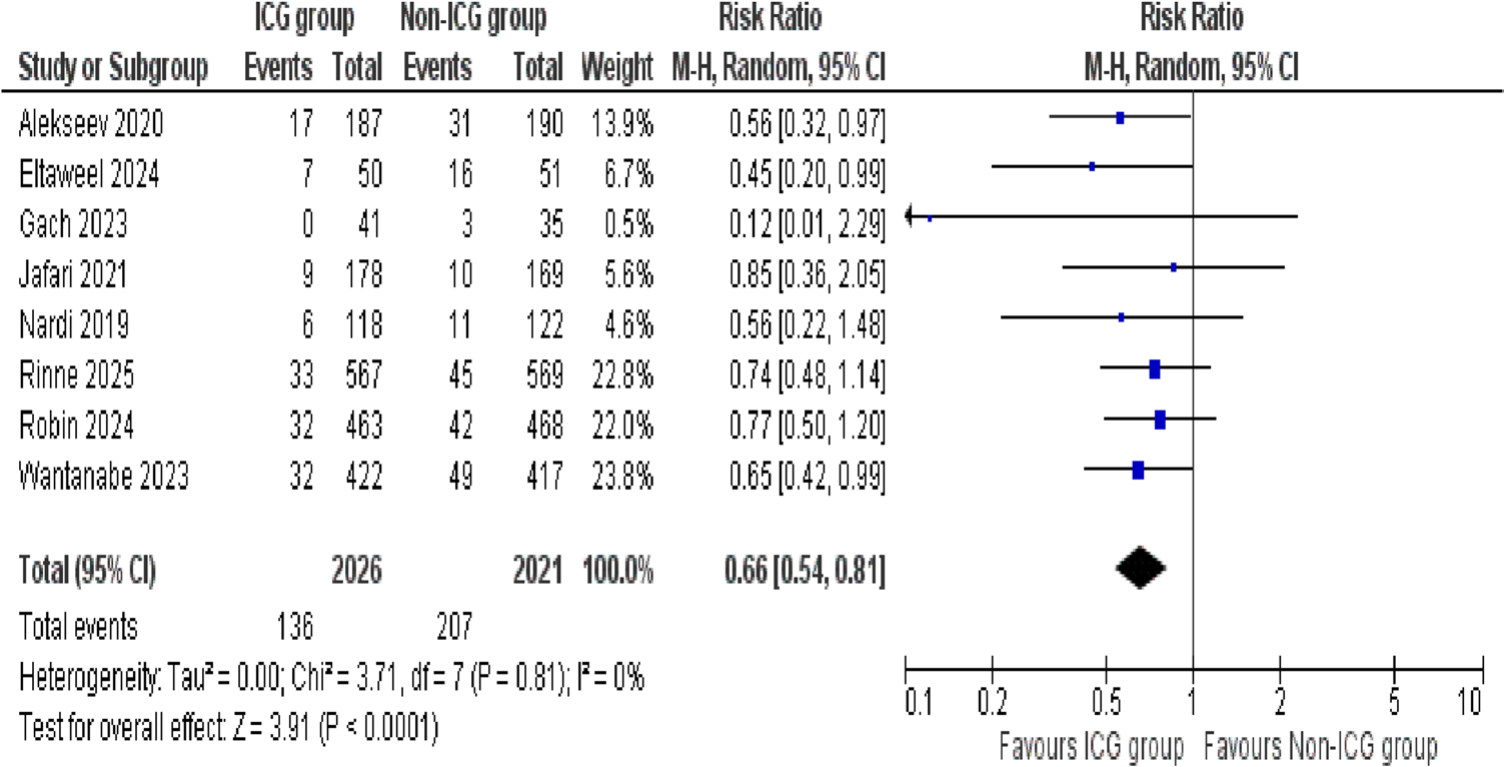
Forest plot of overall anastomotic leak

The outcome of overall anastomotic leak was analyzed by eight studies, with a total sample size of 4047 patients (2026 in the ICG group and 2021 in the non-ICG group). The analysis showed that the use of ICG significantly reduced the risk of anastomotic leaks compared to the non-ICG group making our results statistically significant (risk ratio [RR] = 0.66; 95% confidence interval [CI] 0.54–0.81; *p* < 0.0001). The analysis revealed no significant heterogeneity among the included studies (*I*^2^ = 0%, *p* = 0.81) and suggested that the studies in our analysis yielded remarkably consistent findings (Fig. 2).

##### Anastomotic leakage occurrence

Anastomotic leakage occurrence outcome was divided into three grades based on severity. Three studies with a total of 2154 patients (1065 in the ICG group and 1089 in the non-ICG group) are analyzed.

For Grade A leakage, the analysis revealed that ICG was statistically significant as compared to the non-ICG group (RR = 0.34, 95% Cl 0.16, 0.72; *p* = 0.005). The absence of heterogeneity (*I*^2^ = 0, *p* = 1.00) suggests consistent findings among included studies.

For Grade B leakage, the pooled analysis showed no significant difference between the ICG group and non-ICG group (RR = 0.69, 95% Cl 0.31, 1.52; *p* = 0.36). The analysis revealed a high degree of consistency across studies with no significant heterogeneity detected (*I*^2^ = 0%, *p* = 0.74).

Likewise, for Grade C leakage, the analysis showed no statistically significant difference between the ICG and non-ICG groups (RR = 0.68; 95% CI 0.33–1.39; *p* = 0.29), with substantial heterogeneity (*I*^2^ = 0%, *p* = 0.57) indicating high consistency across studies.

**Fig. 3:**
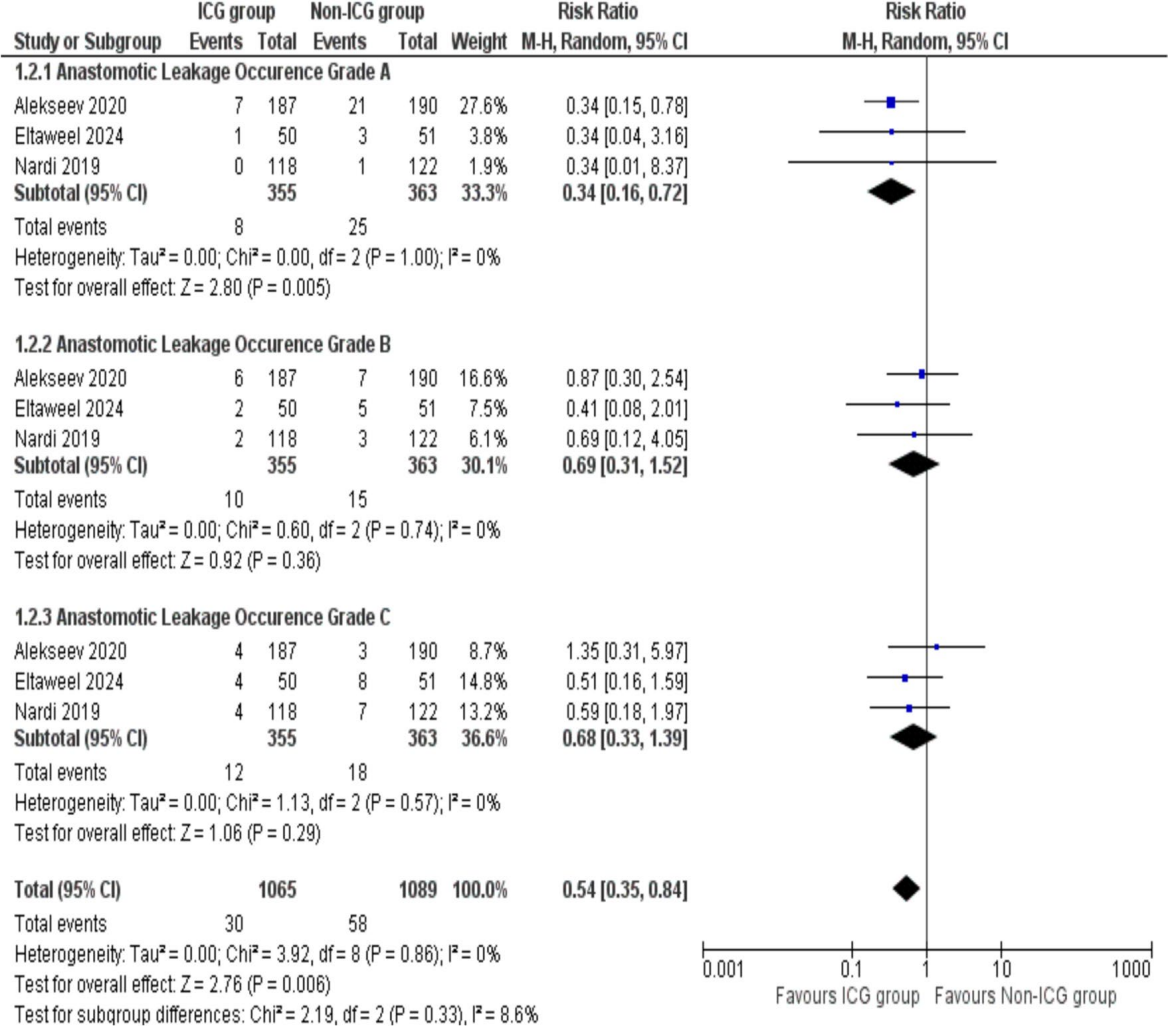
Forest plot of anastomotic leakage occurrence

When considering all grades together, the overall pooled analysis was conducted, which showed that the ICG group had a significantly lower risk of anastomotic leakage compared to the non-ICG group (RR = 0.54; 95% CI 0.35–0.84; *p* = 0.006). Minimal heterogeneity was detected among the included studies (*I*^2^ = 8.6%, *p* = 0.33) pointing a high degree of consistency among included studies (Fig. 3).

##### Clavien–Dindo scale

The Clavien–Dindo scale was utilized to categorize complications into four grades. Grade I was reported by a total of six studies. The analysis revealed a significantly higher difference in the ICG group as compared to the non-ICG group. (RR = 0.67, 95% Cl 0.49, 0.92; *p* = 0.01). No substantial heterogeneity was observed in the study (*I*^2^ = 0%, *p* = 0.86) indicating consistent results.

For Grade II complications, a total of six studies were included. The analysis showed no statistically significant difference between the ICG group and non-ICG group (RR = 1.01, 95% Cl 0.79, 1.28; *p* = 0.97). There was a slight heterogeneity in the analysis (*I*^2^ = 10%, *p* = 0.35) indicating slight variability in studies.

Grade IIIa complications were analyzed across six studies. No statistically significant difference was found in the ICG group as compared to non-ICG group (RR = 0.91, 95% Cl 0.68, 1.23; *p* = 0.54). The studies included showed remarkable consistency, as evidenced by no heterogeneity (*I*^2^ = 0%, *p* = 0.62).

Grade IIIb outcome was assessed by a total of six studies. There was an insignificant difference in the ICG group in comparison to the non-ICG group (RR = 0.81, 95% Cl 0.43, 1.53; *p* = 0.51). Slight heterogeneity was observed in the analysis (*I*^2^ = 30%, *p* = 0.21), which indicated slight variability in studies but it remained within an acceptable range.

**Fig. 4:**
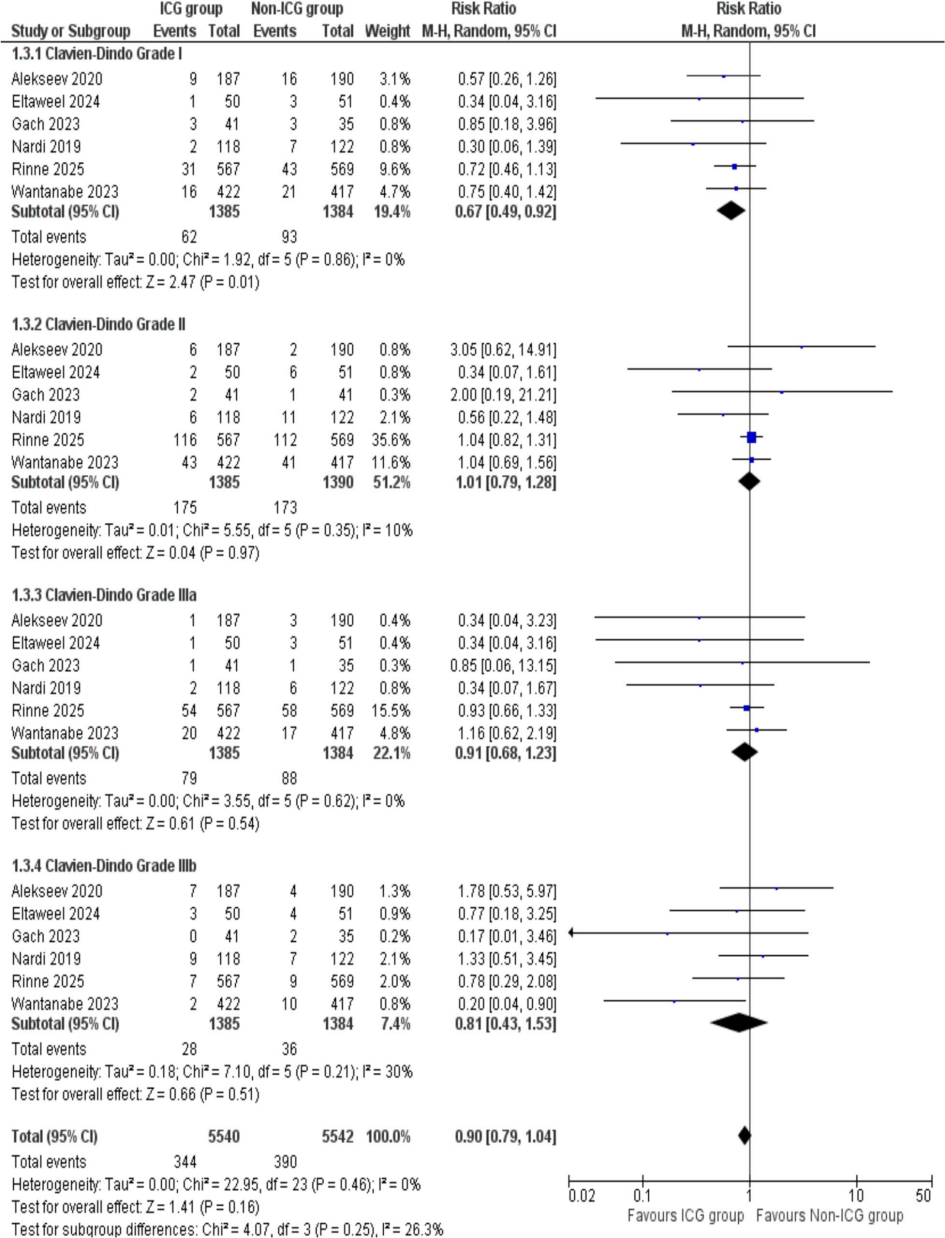
Forest plot of Clavien–Dindo Scale

When combining all grades, the results show no statistically significant difference between the ICG and non-ICG groups, with the overall risk ratio being 0.90 (95% Cl 0.79, 1.04; *p* = 0.16) and no heterogeneity was observed (*I*^2^ = 0%, *p* = 0.46) (Fig. 4).

#### Secondary outcomes

##### Abdominal Bleeding

**Fig. 5:**
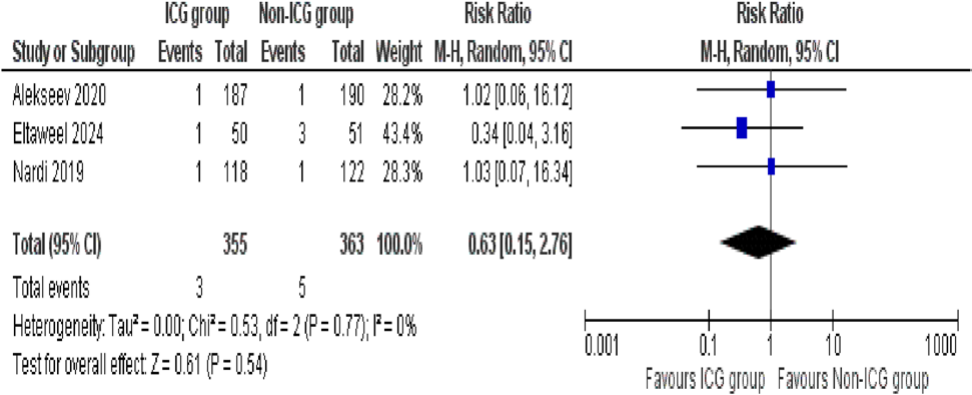
Forest plot of abdominal bleeding

The outcomes of abdominal bleeding were evaluated by three studies. The pooled analysis showed no statistically significant difference between the ICG and non-ICG groups (RR = 0.63; 95% CI 0.15–2.76; *p* = 0.54). The studies included showed remarkable consistency, as evidenced by no heterogeneity (*I*^2^ = 0%, *p* = 0.77) (Fig. 5).

##### Complications

**Fig. 6:**
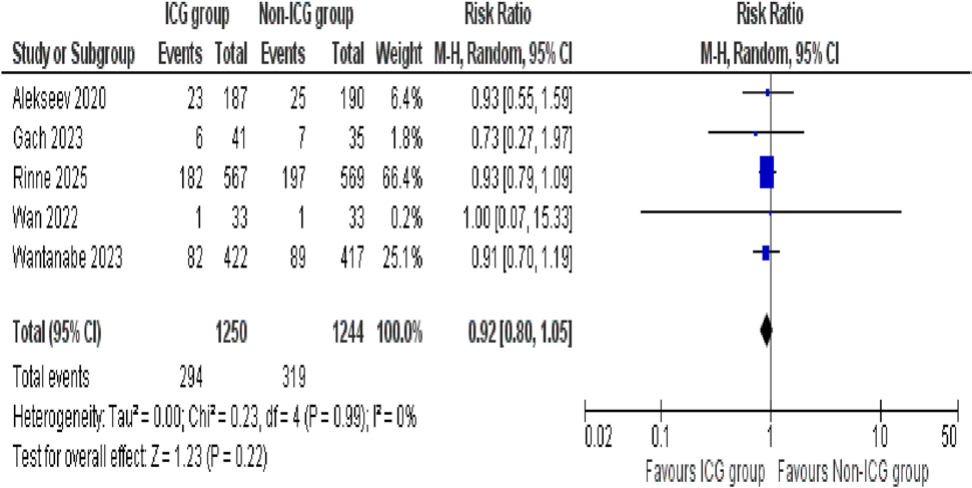
Forest plot of complications

The occurrence of complications between the ICG and non-ICG groups is evaluated by five studies. The pooled analysis showed no statistically significant difference between the two groups (RR = 0.92; 95% CI 0.80–1.05; *p* = 0.22). Importantly, the analysis revealed a high degree of consistency across studies, as no substantial heterogeneity was observed (*I*^2^ = 0%, *p* = 0.99) (Fig. 6).

##### Mechanical ileus

**Fig. 7:**
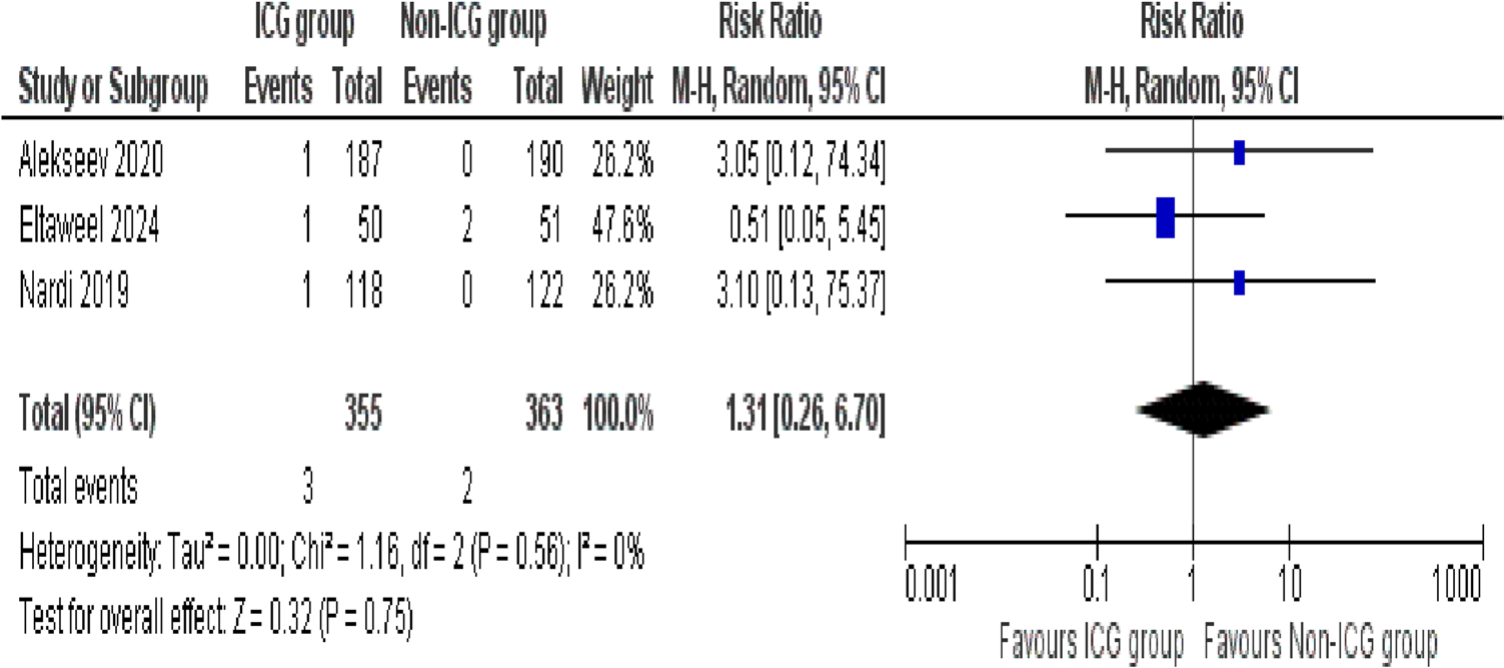
Forest plot of mechanical ileus

A total of three studies assessed the occurrence of mechanical ileus between both the groups. The pooled analysis showed no statistically significant difference between the ICG and Non-ICG groups (RR = 1.31; 95% CI: 0.26–6.70; *p* = 0.75). No heterogeneity was observed during analysis (*I*^2^ = 0%, *p* = 0.56), indicating high consistency across studies (Fig. 7).

##### Paralytic ileus

**Fig. 8:**
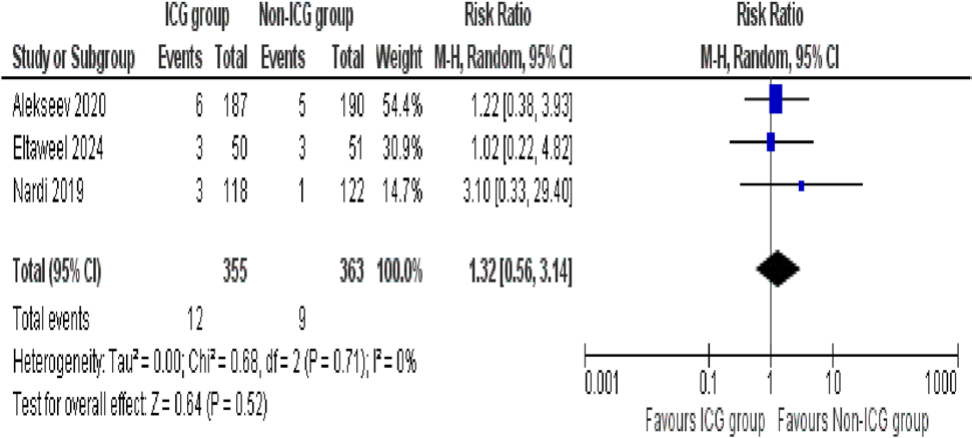
Forest plot of paralytic ileus

The occurrence of paralytic ileus is accessed by a total of three studies between the ICG and non-ICG groups. The pooled analysis showed no significant difference between the groups (RR = 1.32; 95% CI: 0.56–3.14; *p* = 0.52). The analysis revealed excellent consistency across the studies, as no substantial heterogeneity was observed (*I*^2^ = 0%, *p* = 0.71) (Fig. 8).

##### Wound infection

**Fig. 9:**
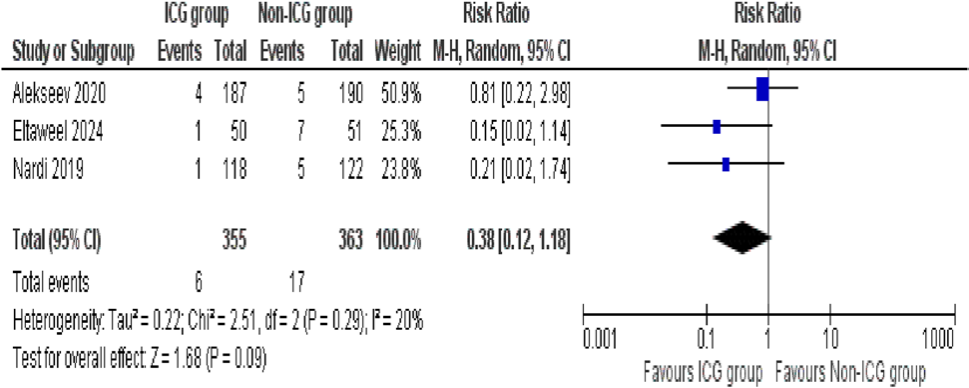
Forest plot of wound infection

Considering the occurrence of wound infection rates, three studies compared the outcomes between the ICG and non-ICG groups. The pooled analysis showed that the ICG group demonstrated a trend toward fewer wound infections, but the difference was not statistically significant (RR = 0.38; 95% CI 0.12–1.18; *p* = 0.09). The results across the studies indicated low heterogeneity, although it was not zero (*I*^2^ = 20%, *p* = 0.29) (Fig. 9).

##### Operating time

**Fig. 10:**
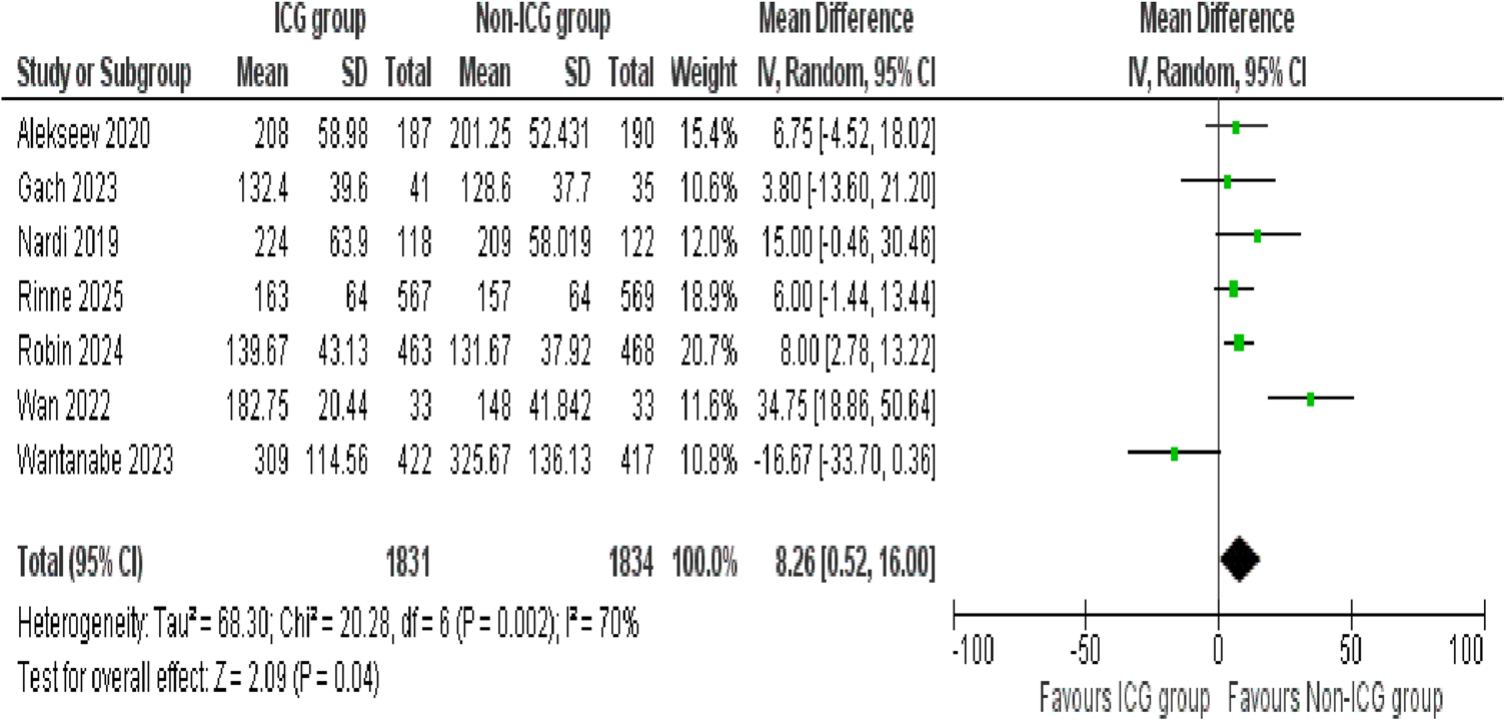
Forest plot of operating time

A total of seven studies compared the operating time between the ICG group and the non-ICG group. The pooled analysis showed that the ICG group had a significantly longer operating time compared to the non-ICG group (MD = 8.26 min, 95% CI 0.52 to 16.00; *p* = 0.04). However, a considerable heterogeneity was observed among the included studies (*I*^2^ = 70%, *p* = 0.002), pointing towards considerable variability in results across studies (Fig. 10).

##### Post operative hospital stay

**Fig. 11:**
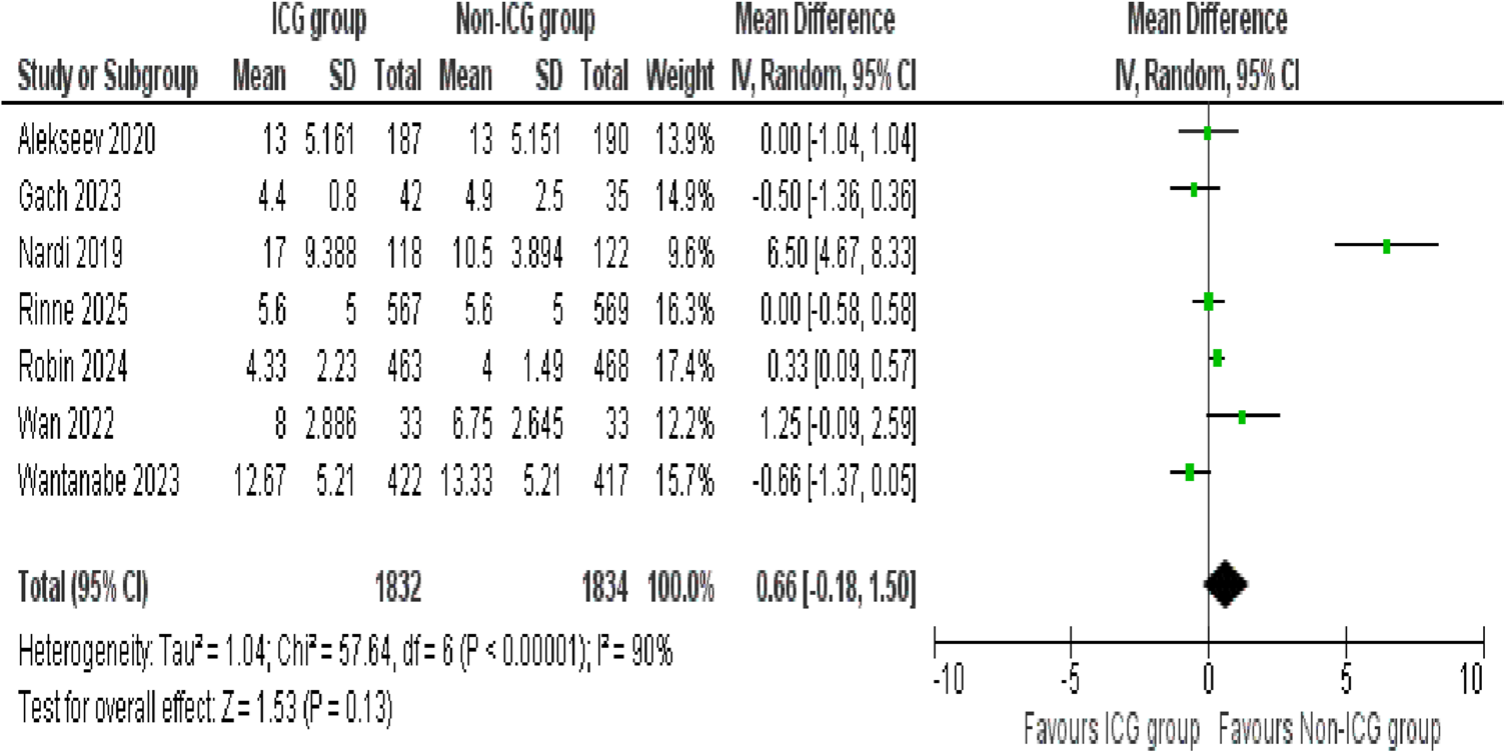
Forest plot of post-operative hospital stay

A total of seven studies assessed the outcome of postoperative hospital stay between the ICG and non-ICG groups. The pooled analysis demonstrated no statistically significant difference between the two groups (mean difference = 0.66; 95% CI − 0.18 to 1.50; *p* = 0.13). A noticeable level of heterogeneity was observed among the included studies (*I*^2^ = 90%, *p* < 0.00001), indicating high variability in the results (Fig. 11).

### Sensitivity analysis

#### Operating time

Considering the substantial heterogeneity identified in the initial operating time analysis, a sensitivity analysis was conducted to address it. Two studies, Wan 2022 and Wantanabe 2023, were excluded due to their significant contribution in overall heterogeneity. A total of five studies were included, which revealed a statistically significant difference favoring the non-ICG group (mean difference = 7.56; 95% CI 3.79 to 11.34; *p* < 0.0001). Notably, after the sensitivity analysis, no heterogeneity was detected among the included studies (*I*^2^ = 0%, *p* = 0.86), indicating high consistency across the findings (Supplementary Figure S2).

#### Post-operative hospital stay

Taking into account the substantial heterogeneity observed in the initial analysis of postoperative hospital stay, a sensitivity analysis was performed to address this. Four studies, Gach (2023), Nardi (2019), Wan (2022), and Wantanabe (2023), which contributed to the variability, were excluded. The analysis included three studies and showed a statistically significant difference, indicating that the ICG group had a slightly longer postoperative hospital stay compared to the non-ICG group (mean difference = 0.27; 95% CI 0.05 to 0.49; *p* = 0.02). Importantly, no evidence of heterogeneity was observed after the sensitivity analysis (*I*^2^ = 0%, *p* = 0.52), indicating strong consistency across the included studies (Supplementary Figure S3).

#### Wound infection

Taking into account the substantial heterogeneity observed in the initial analysis of postoperative hospital stay, a sensitivity analysis was performed to address this. The study Alekseev (2020) was identified as a contributor to variability and was excluded. The analysis included three studies and showed that the ICG group had a significantly lower risk of wound infections compared to the non-ICG group (risk ratio = 0.17; 95% CI 0.04 to 0.76; *p* = 0.02). Importantly, no evidence of heterogeneity was observed after the sensitivity analysis (*I*^2^ = 0%, *p* = 0.82), indicating strong consistency across the included studies (Supplementary Figure S4).

## Discussion

This meta-analysis aims to evaluate the impact of indocyanine green (ICG) fluorescence imaging on surgical outcomes compared to conventional (non-ICG) methods, focusing on both primary and secondary outcomes across multiple studies. By extracting the data from nine clinical trials, we analyze whether the use of ICG fluorescence imaging is associated with improved intraoperative and postoperative outcomes or not. Our findings offer a detailed picture of the role of ICG in enhancing patient outcomes and improving intraoperative decision making.

For our primary outcomes, the pooled analysis demonstrates a significant reduction in overall anastomotic leakage with the use of ICG compared to the non-ICG group, with minimal heterogeneity (*I*^2^ = 8.6%). Considering different grades of leakage, the most beneficiary results are driven by the significant reduction in Grade A leaks, while Grades B and C did not show statistically significant differences. These findings indicate advanced technologies and intraoperative techniques, such as ICG fluorescence–guided surgery, should be prioritized to improve outcomes [30]. The Clavien–Dindo scale, which is used to classify postoperative complications, also revealed no significant difference between both the ICG and non-ICG groups, suggesting it may not significantly lower the severity or frequency of clinically relevant postoperative complications, supported by other studies as well [31]. The use of ICG fluorescence imaging significantly reduces the overall anastomotic leak rates, with no prominent sign of heterogeneity across studies, emphasizing its potential to improve anastomotic integrity in surgical procedures.

Among the secondary outcomes, abdominal bleeding was observed less frequently in the ICG group compared to non ICG group, although this difference did not reach statistical significance. This trend indicates that ICG may help reduce the bleeding, likely by enhancing the ability to visualize the vascular anatomy more clearly as supported by several studies [32]. Similarly wound infection rates also showed a lower relative risk in the ICG group (RR = 0.38), but this difference was not statistically significant.

In terms of postoperative complications, the analysis showed no significant difference between the ICG group and non-ICG group. The risk ratio was 0.92, and the heterogeneity across studies was negligible, suggesting the results were consistent. For bowel-related complications, both the mechanical and paralytic ileus showed no significant differences between groups. Mechanical ileus had a risk ratio of 1.32, while paralytic ileus showed similar non-significant results with an RR of 1.31. Overall, these findings indicate ICG does not appear to have a meaningful impact on the risk of developing postoperative ileus in either form.

Similar results were reported by previous literature also, evaluating the impact of ICG fluorescence imaging on surgical outcomes. Deng et al. (2022) analyzed the safety and efficacy of ICG guided lymph nodes dissection and found that although ICG significantly improved intraoperative parameters, such as reducing blood loss and increasing lymph node yield, it did not lead to a significant reduction in overall postoperative complications [33]. Likewise, Keller et al. (2021) investigated the use of ICG fluorescence in colorectal surgeries and reports that while it contributed to a lower rate of anastomotic leakage, it did not significantly alter the risk of other postoperative complications, including the incidence of mechanical or paralytic ileus [34] (These studies support our findings as well, which also showed that ICG use did not significantly lower the rate of overall postoperative complications or bowel-related issues. Undoubtedly, ICG is useful in enhancing intraoperative visualization and precision; it does not seem to have a major impact on postoperative complications.

Interestingly, one of the few outcomes that show a statistically significant difference was operating time. Patients in the ICG group experienced a significant increase in operating duration (mean difference = 8.26 min). This small increase could be due time required to administer and interpret fluorescence imaging intraoperatively. However, our initial analyses demonstrate a significant heterogeneity (*I*^2^ = 70%), suggesting the variability across the studies. To address this, a sensitivity analysis was conducted by excluding studies contributing to heterogeneity. Sensitivity analysis confirmed a consistent and statistically significant increase in operating time (mean difference = 7.56 min), now with no observed heterogeneity (*I*^2^ = 0%). These findings support the evidence that ICG results in increase in surgical duration, independent of variability between studies. This slight increase in operative time is likely acceptable, considering the clinical advantages of enhanced tissue perfusion assessment and intraoperative visualization offered by ICG imaging [35–37]. In contrast, no statistically significant difference is noticed in postoperative hospital stay between the ICG and non-ICG groups, with a mean difference of 0.66 days. However, the results show substantial heterogeneity likely due to variations in institutional discharge policies, perioperative care protocols, and patient populations [38]. To account for this, we conducted sensitivity analysis, excluding studies contributing for heterogeneity. Following this, the mean difference was reduced to 0.27 days, reaching statistical significance. ICG use is associated with a small but statistically significant increase in postoperative hospital stay.

Including these as secondary outcomes is important because they provide a broader assessment of surgical safety and recovery beyond primary outcomes such as anastomotic leakage occurrence and the Clavien–Dindo scale. While these primary outcomes are essential, they mainly reflect direct surgical success and may not capture the complete picture. Secondary outcomes offer valuable insight into perioperative risks, patient morbidity, and healthcare resource utilization—critical factors in optimizing surgical protocols and improving patient outcomes in colorectal surgery. They also help evaluate the clinical impact of interventions, guide perioperative decision-making, and assess the overall quality of surgical care. Tracking these parameters allows us to gain deeper insight into the role of ICG in colorectal surgery and enhances the relevance and clinical applicability of our findings. Moreover, several previous meta-analyses have also included these outcomes in their analyses [33, 39].

In summary, while ICG-guided surgery shows promise in enhancing intraoperative visualization and potentially reducing certain complications like bleeding and wound infection, our analysis does not demonstrate statistically significant improvements in the most critical surgical outcomes, such as anastomotic leakage and postoperative complication severity. The slight increase in operating time appears to be the only consistent and significant difference. These results suggest that while ICG is a valuable adjunct tool, its impact on clinical outcomes remains modest and may depend on institutional protocols and surgeon expertise. Further high-quality randomized controlled trials with standardized reporting are necessary to establish clearer evidence for its routine use. ICG-guided surgery seems to be a helpful technique for improving outcomes during operations, but its s broader impact on postoperative recovery and long-term clinical benefit maybe limited and vary depending on the situation.

Besides ICG fluorescence imaging, several other techniques are utilized in GIT surgery to enhance patient’s outcomes and intraoperative precision. Near-infrared (NIR) imaging is particularly used for enhancing intraoperative visualization and improving surgical precision. It is often combined with indocyanine green (ICG) to assess real-time tissue perfusion, aiding in the assessment of anastomotic integrity. This helps reduce the risk of complications such as anastomotic leakage by ensuring adequate blood flow [40, 41]. Doppler ultrasound is another widely used method for enhancing intraoperative decision-making by assessing blood flow during surgical procedures. It is mainly used for evaluating vascular integrity and perfusion at the anastomotic site, helping surgeons ensure adequate blood supply before completing the connection [42]. Robot-assisted surgery has also become an increasingly popular approach due to its ability to enhance precision, dexterity, and visualization. Using these advanced systems, surgeons can perform complex tasks through minimally invasive techniques, and this approach also allows for greater accuracy in dissection and suturing [43].

In addition, intraoperative endoscopy is frequently used to inspect the anastomosis for bleeding and leakage, which allows identifying technical issues immediately, enabling timely correction during surgery. We have other techniques as well, such as air leak testing, which involves filling the bowel with air or CO₂, while the area with the anastomosis is submerged in saline. Although this is a simple technique, it is highly effective in detecting leaks intraoperatively. Other strategies may include fluorescence angiography with alternative dyes such as with methylene blue or fluorescein, which has also been investigated. Besides the availability of many techniques, ICG remains the most widely used and effective approach, primarily due to its excellent safety profile and superior imaging quality.

### Limitations

While this meta-analysis offers valuable insights, it is not without limitations. One key issue is the heterogeneity noted in several secondary outcomes, most notably postoperative hospital stay and operating time, which may reflect differences in institutional protocols, patient populations, and perioperative care, thereby limiting the generalizability of the results. Although all included studies were randomized controlled trials, discrepancies in study design, sample size, and surgical experience may still introduce some degree of bias. Additionally, the lack of uniform protocols for indocyanine green (ICG) administration and fluorescence interpretation across studies could affect the consistency of outcome reporting. The decision to exclude observational studies also narrows the perspective, potentially overlooking data from real-world clinical settings. Despite conducting sensitivity analyses to address variability, the possibility of residual confounding cannot be entirely excluded. Finally, the focus on short-term surgical outcomes leaves unanswered questions about the long-term impact of ICG fluorescence imaging on anastomotic healing and patient recovery, highlighting the need for future research with extended follow-up durations.

### Future implications

The findings of this meta-analysis underscore the clinical value of integrating indocyanine green (ICG) fluorescence imaging into surgical practice, particularly for its role in reducing anastomotic leak rates. While this technology did not demonstrate a statistically significant impact on most secondary outcomes, the reduction in Grade A leaks and the trend toward fewer wound infections and less abdominal bleeding suggest that ICG offers meaningful intraoperative advantages, especially in enhancing vascular visualization and anastomotic integrity.

Looking ahead, further research is warranted to better define the patient populations and surgical contexts where ICG provides the greatest benefit. Future randomized controlled trials with larger sample sizes and standardized protocols are essential to explore ICG’s long-term impact on postoperative outcomes, cost-effectiveness, and patient-reported recovery metrics. Additionally, advances in fluorescence technology and imaging platforms may further refine its utility, potentially improving its integration into minimally invasive and robotic-assisted surgeries.

Moreover, the learning curve and logistical demands associated with ICG use—such as longer operating times—highlight the importance of surgical training and workflow optimization. Health systems adopting ICG should consider structured implementation strategies to minimize procedural delays while maximizing clinical benefit. As technology and surgeon familiarity evolve, these procedural inefficiencies may diminish over time.

## Conclusion

This comprehensive meta-analysis demonstrates that the use of ICG fluorescence imaging during surgery is associated with a significant reduction in overall anastomotic leakage, particularly for Grade A leaks. These results were consistent across studies with minimal heterogeneity, reinforcing the reliability of this finding. While ICG did not significantly alter the incidence of other postoperative complications—including mechanical and paralytic ileus, bleeding, and infection—its potential to enhance intraoperative decision-making is evident.

Although an increase in operating time was observed, this modest extension is arguably a reasonable trade-off for improved anastomotic outcomes. Importantly, the results suggest that while ICG does not universally prevent postoperative complications, it serves as a valuable adjunct in high-stakes phases of surgery, particularly where tissue perfusion is critical.

In conclusion, ICG fluorescence imaging represents a promising tool that enhances surgical precision and safety. Its targeted use in appropriate clinical scenarios could contribute to better short-term outcomes without significantly increasing risk. As the field of fluorescence-guided surgery continues to evolve, future studies should aim to refine its application and evaluate its broader impact on surgical quality and patient care.

## Supplementary Information

Below is the link to the electronic supplementary material.

ESM1 (DOCX 1.18 MB)

## Data Availability

All data generated or analyzed during this study are included in this published article (and its supplementary files). The datasets used are available from the corresponding author upon reasonable request.
