# Supplementary material for: Efficacy and safety of indocyanine green fluorescence imaging in colorectal cancer: a systematic review and meta-analysis of randomized controlled trials
Source: Int J Colorectal Dis. 2025 Sep 10;40(1):193. doi: 10.1007/s00384-025-04941-7 (PMC12420736; doi:10.1007/s00384-025-04941-7)
Supplement: Supplementary file 1 — (DOCX 1.18 MB) [file 384_2025_4941_MOESM1_ESM.docx]

| **Primary Outcomes** | **Outcome** | **Definition** |
| --- | --- | --- |
| 1 | Overall Anastomotic  Leak | Any type of leakage at the bowel connection site after colorectal cancer surgery, regardless of severity; used to assess whether ICG imaging helps prevent this complication.. |
| 2 | Anastomotic Leakage Occurrence | The presence or absence of a leak at the anastomosis site; measures the effectiveness of ICG in reducing leak incidence |
| 3 | Clavien-Dindo Scale | A classification system used to grade the severity of postoperative complications; helps evaluate how ICG use impacts the seriousness of surgical outcomes.. |
| **Secondary Outcomes** | **Outcome** | **Definition** |
| 1 | Abdominal Bleeding | Bleeding within the abdominal cavity after surgery; assessed to determine if ICG reduces vascular injuries and bleeding risk.. |
| 2 | Complications | Any adverse events occurring after colorectal surgery, such as infections or leaks; used to evaluate the overall safety of ICG-guided surgery. |
| 3 | Mechanical Ileus | A blockage in the intestines due to physical obstruction; tracked to see if ICG use indirectly reduces such risks. |
| 4 | Paralytic Ileus | A temporary loss of bowel movement without blockage, often due to surgical stress; analyzed to assess recovery and bowel function post-ICG use. |
|  |  |  |
| 5 | Wound Infection | Infection at the surgical incision site; used to examine if ICG guidance contributes to lower infection rates. |
| 6 | Operating Time | The total time taken for colorectal surgery; measured to assess whether ICG imaging increases or decreases surgery duration. |
| 7 | Postoperative Hospital Stay | Number of days a patient stays in the hospital after surgery; used to evaluate if ICG use leads to faster recovery and discharge. |

**Supplementary Table 1 : Definition of Outcomes**

| **Summary of findings:** | | | | | | |
| --- | --- | --- | --- | --- | --- | --- |
| **Indocyanine Green Fluorescence Imaging compared to placebo in colorectal cancer** | | | | | | |
| **Patient or population:** colorectal cancer  **Setting:**  **Intervention:** Indocyanine Green Fluorescence Imaging  **Comparison:** placebo | | | | | | |
| Outcomes | **Anticipated absolute effects^*^** (95% CI) | | Relative effect (95% CI) | № of participants (studies) | Certainty of the evidence (GRADE) | Comments |
|  | **Risk with placebo** | **Risk with Indocyanine Green Fluorescence Imaging** |  |  |  |  |
| Overall anastomotic leak assessed with: RR | 102 per 1,000 | **68 per 1,000** (55 to 83) | **RR 0.66** (0.54 to 0.81) | 4047 (8 RCTs) | ⨁⨁⨁⨁ High |  |
| Clavien-Dindo Scale assessed with: RR | 70 per 1,000 | **63 per 1,000** (56 to 73) | **RR 0.90** (0.79 to 1.04) | 11082 (6 RCTs) | ⨁⨁⨁◯ Moderate^a^ |  |
| anastomotic leakage occurence assessed with: RR | 53 per 1,000 | **29 per 1,000** (19 to 45) | **RR 0.54** (0.35 to 0.84) | 2154 (3 RCTs) | ⨁⨁⨁⨁ High |  |
| wound infection assessed with: RR | 47 per 1,000 | **18 per 1,000** (6 to 55) | **RR 0.38** (0.12 to 1.18) | 718 (3 RCTs) | ⨁⨁⨁◯ Moderate^a^ |  |
| Post operative hospital stay assessed with: MD | The mean post operative hospital stay was **0** | **0**  (0 to 0 ) | - | 3666 (7 RCTs) | ⨁⨁◯◯ Low^a,b^ |  |
| Paralytic ileus assessed with: RR | 25 per 1,000 | **33 per 1,000** (14 to 78) | **RR 1.32** (0.56 to 3.14) | 718 (3 RCTs) | ⨁⨁⨁◯ Moderate^a^ |  |
| Operating time assessed with: MD | The mean operating time was **0** | **0**  (0 to 0 ) | - | 3665 (7 RCTs) | ⨁⨁◯◯ Low^a,b^ |  |
| Mechanical ileus assessed with: RR | 6 per 1,000 | **7 per 1,000** (1 to 37) | **RR 1.31** (0.26 to 6.70) | 718 (3 RCTs) | ⨁⨁⨁◯ Moderate^a^ |  |
| Complications assessed with: RR | 256 per 1,000 | **236 per 1,000** (205 to 269) | **RR 0.92** (0.80 to 1.05) | 2494 (5 RCTs) | ⨁⨁⨁⨁ High |  |
| Abdominal bleeding assessed with: RR | 14 per 1,000 | **9 per 1,000** (2 to 38) | **RR 0.63** (0.15 to 2.76) | 718 (3 RCTs) | ⨁⨁⨁◯ Moderate^a^ |  |
| ***The risk in the intervention group** (and its 95% confidence interval) is based on the assumed risk in the comparison group and the **relative effect** of the intervention (and its 95% CI).  **CI:** confidence interval; **RR:** risk ratio | | | | | | |
| **GRADE Working Group grades of evidence** **High certainty:** we are very confident that the true effect lies close to that of the estimate of the effect. **Moderate certainty:** we are moderately confident in the effect estimate: the true effect is likely to be close to the estimate of the effect, but there is a possibility that it is substantially different. **Low certainty:** our confidence in the effect estimate is limited: the true effect may be substantially different from the estimate of the effect. **Very low certainty:** we have very little confidence in the effect estimate: the true effect is likely to be substantially different from the estimate of effect. | | | | | | |

#### Explanations

a. Wide confidence interval crossing the line of null effect

b. High heterogeniety

**Supplementary Table S2:** Grade Assessment


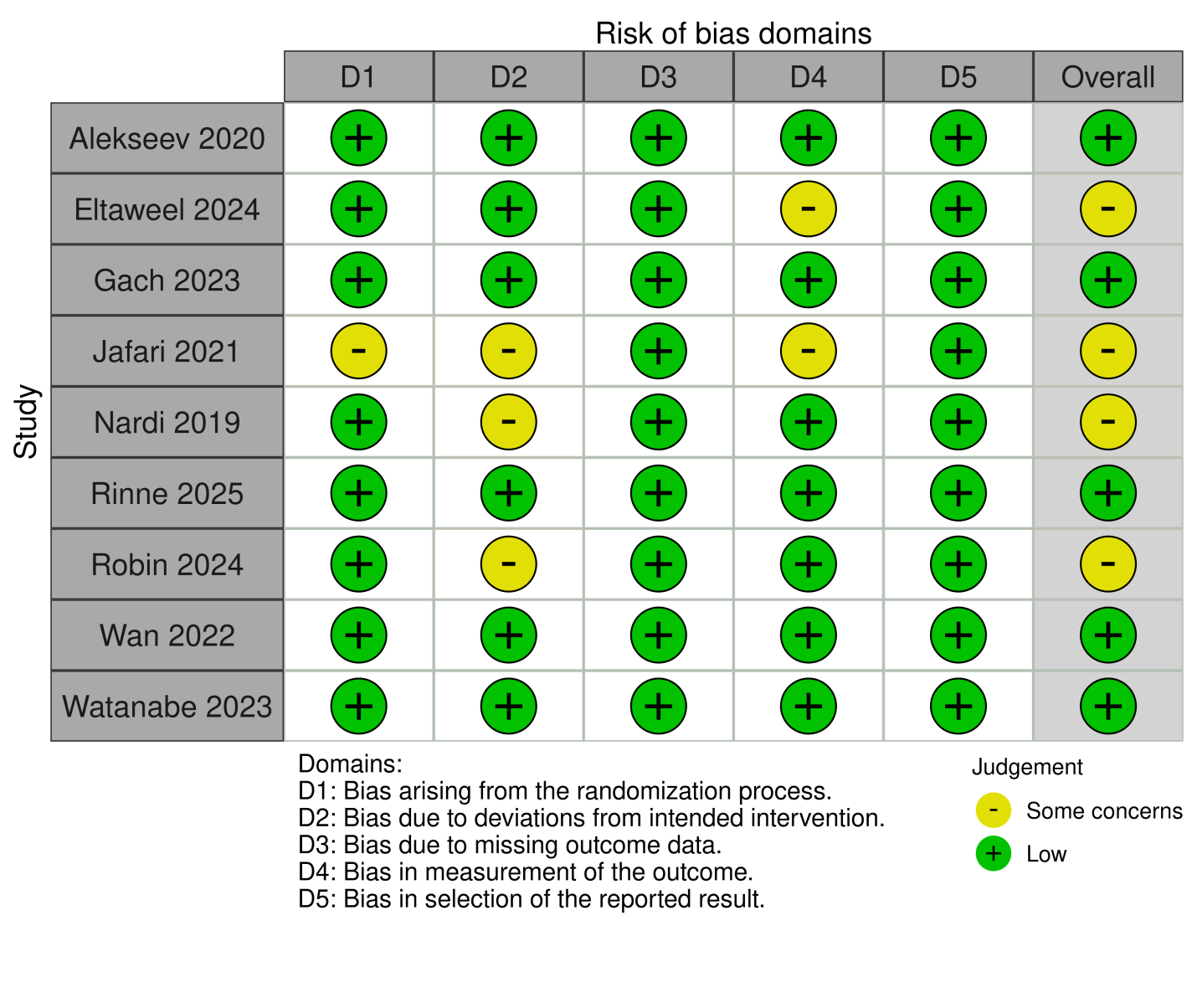


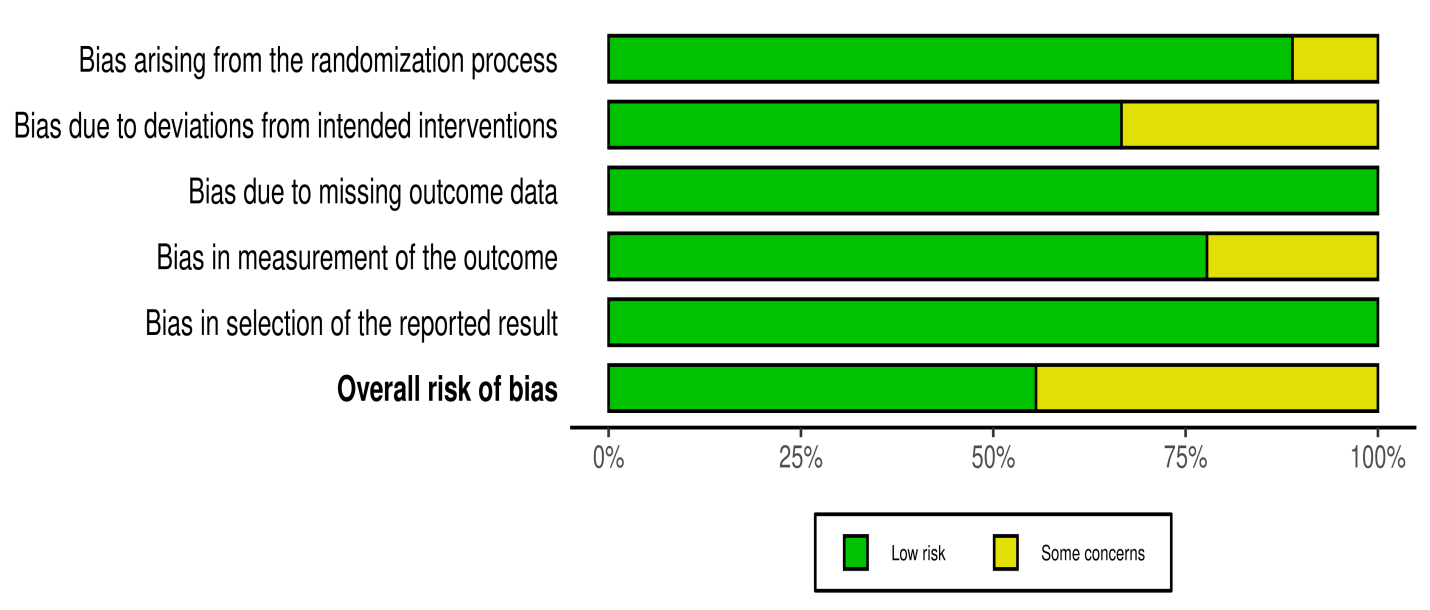


**Supplementary Figure 1: Risk of Bias Assessment**


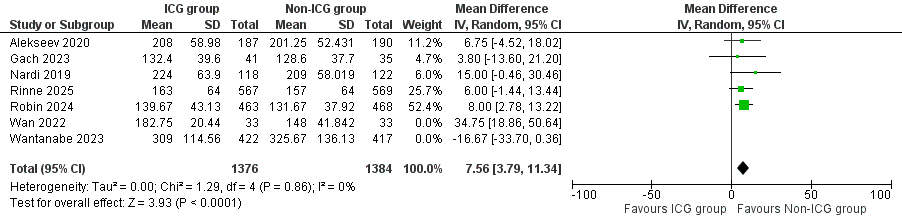


**Fig. S2 :** Forest plot of Operating time after removal of Wan 2022 and Watanabe 2023


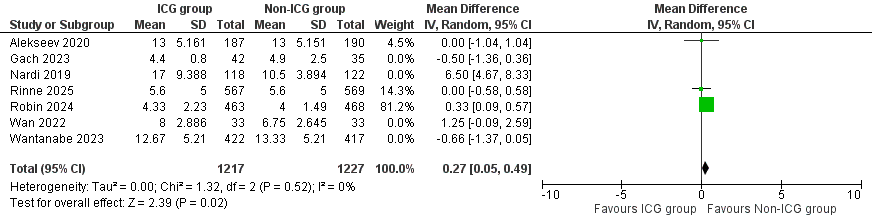


**Fig. S3 :** Forest plot of Post Operative Hospital Stay after Sensitivity Analysis


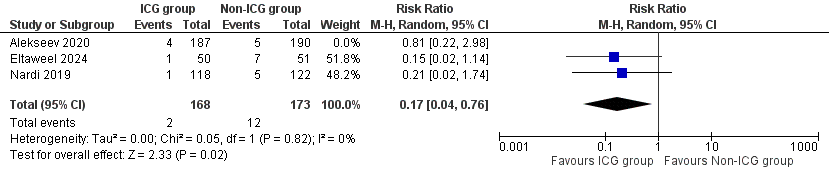


**Fig. S4 :** Forest plot of wound Infection after removing Alekseev 2020
